# Visual Sensor Based Abnormal Event Detection with Moving Shadow Removal in Home Healthcare Applications

**DOI:** 10.3390/s120100573

**Published:** 2012-01-05

**Authors:** Young-Sook Lee, Wan-Young Chung

**Affiliations:** 1 Electronic Information Communication Research Center, Pukyong National University, Busan 608-737, Korea; E-Mail: yulisis@pknu.ac.kr; 2 Department of Electronic Engineering, Pukyong National University, Busan 608-737, Korea

**Keywords:** abnormal event detection, visual sensor, shape features variation, shadow removal algorithm, ubiquitous healthcare surveillance

## Abstract

Vision-based abnormal event detection for home healthcare systems can be greatly improved using visual sensor-based techniques able to detect, track and recognize objects in the scene. However, in moving object detection and tracking processes, moving cast shadows can be misclassified as part of objects or moving objects. Shadow removal is an essential step for developing video surveillance systems. The goal of the primary is to design novel computer vision techniques that can extract objects more accurately and discriminate between abnormal and normal activities. To improve the accuracy of object detection and tracking, our proposed shadow removal algorithm is employed. Abnormal event detection based on visual sensor by using shape features variation and 3-D trajectory is presented to overcome the low fall detection rate. The experimental results showed that the success rate of detecting abnormal events was 97% with a false positive rate of 2%. Our proposed algorithm can allow distinguishing diverse fall activities such as forward falls, backward falls, and falling asides from normal activities.

## 1. Introduction

The world is experiencing a rapid increase in the number of people living to an advanced old age. Thus, the global population is aging, because people older than 60 are the fastest growing population sector in all countries. This increase can be attributed to advances in science, technology and medicine, which have led to reductions in infant and maternal mortality, infectious and parasitic diseases, occupational safety measures, and improvements in nutrition and education. We need to develop intelligent healthcare surveillance applications that provide a more intelligent and inexpensive way of managing care for older people and patients suffering from age-related chronic illnesses, such as physical disorders, that require continuous, long-term monitoring rather than episodic assessments [1].

Abnormal events, such as unexpected falls, are one of the most complex and costly health care problems facing the elderly. A fall is defined as a sudden, uncontrolled and unintentional, downward displacement of the body to the ground or another object, excluding falls resulting from violent blows or other intentional actions [2]. Falls can result in serious injuries such as joint dislocations, fractures, cerebral hemorrhage, severe head injuries, or even death. Falls are the most frequent accidents in the world, exceeding both traffic accidents and fires, and they are a major cause of disability, as well as the leading cause of mortality due to injury in people aged over sixty-five [3].

Most fall accidents affect the elderly living alone and usually occur at home. Automatic sensor-based fall detection techniques have been developed to minimize fall-related injuries of older people, and these can be classified into three main categories of ubiquitous healthcare monitoring applications [4,5], *i.e.*, wearable sensor-based, acoustic sensor-based, and visual sensor-based methods. The disadvantage of wearable sensor-based fall detection algorithms is that they cannot detect falls when older people forgot to wear detectors. Acoustic sensor–based systems use microphones to detect falls by analyzing the frequency components of vibrations produced by the impacts of human body parts on the ground. However, these systems only provide crude data that are difficult to interpret.

In contrast, visual sensor-based systems provide two main advantages. One is that vision sensor-based techniques provide easy-to-interpret data. The other is not necessary to wear any devices to detect fall events. Visual sensor-based methods have been extensively researched because of their accuracy, robustness, and non-intrusiveness. Researchers have recently developed vision sensor-based systems for detecting fall events. However, these systems often have low accuracy and high false alarm rates.

Nait-Charif and Mckenna [6] tracked subjects using a coarse ellipse in a supportive home environment, and analyzed the resulting trajectory to detect inactivity outside the normal zones of inactivity like chairs or sofas. However, this method used a surveillance camera sensor that was unsuitable for distinguishing fall activities from normal daily activities, such as sitting down rapidly. Lee and Mihailidis [7] detected a fall by analyzing the shape of the subject’s silhouette and 2-D velocity of the person, with special thresholds for inactivity zones like the bed. SIMABD (Smart Inactivity Monitor using Array-Based Detectors) used a low-cost array of infrared detectors to capture a low-resolution blurry image and a neural network that classified falls based on vertical velocity [8]. However, this method had a very low fall detection rate. The fall detector’s classifier performed poorly, because only vertical velocity was used as an input for the classifier. Recently, a hybrid human fall detection scheme was presented [9]. This fall detection system is based on an ingenious combination of skeleton feature and human shape variation. This method showed a low detection rate. Other work has been done using multi-camera systems [10,11]. These systems have several problems. One problem is that these methods need to employ more than four cameras to detect falls. In real living environments, these systems are not easy to use. Another problem is that test image sequences of each camera need to be synchronized, which makes the algorithm more difficult to implement than a monocular camera one.

To improve the fall detection rate and increase the system reliability, we propose a novel approach based on shape feature variation and 3-D depth information for detecting unexpected falls of the elderly in real-time home healthcare surveillance settings. The system allows detection of various types of unexpected falls such as forward falls, backward falls, and falling sideways. Our proposed shadow removal method is essential for detecting, tracking, and identifying a person in the scene and is helpful for reducing false alarms.

## 2. The Approach

The primary goal of this paper is to design a novel computer vision technique that can discern abnormal behavior from normal activities based on shape features variation and 3-D depth information. Our abnormal event detection method for home healthcare surveillance systems is divided into three major parts: foreground detection with shadow removal, object tracking, and abnormal event detection. Each part of the approach is described in greater detail below.

### 2.1. Foreground Detection with Shadow Removal

The first stage in our abnormal event detection algorithm is the segmentation of the person from the background [12,13]. A foreground model based on traditional background subtraction is unsuitable for extracting the objects of interest, because it cannot continue to track target objects if the foreground in the scene does not move. To address this problem, we combined GMM (Gaussian mixture model) and weighted subtraction between consecutive difference images in order to segment a moving object of interest as the foreground [14].

Shadow removal is a major challenge in many computer vision-based automatic applications, such as healthcare monitoring systems, video surveillance systems, obstacle tracking/recognition, and intelligent transportation systems. Moving cast shadows can be misclassified as object parts or moving objects during foreground extraction, because the cast shadow region may be included in the moving object region after the segmentation process. It is difficult to separate shadow regions from moving objects. Thus, moving shadows create serious problems, such as object merging, object shape deformation, and object losses.

To address these problems, we propose a reliable and accurate algorithm for object detection and shadow removal. Our proposed algorithm can detect and eliminate cast shadows by exploiting their spectral properties. The cast shadow defines the area projected onto the scene by an object, and it can be divided into the umbra and the penumbra. Shadow removal algorithms need to eliminate all the cast shadow effects, including the umbra and penumbra. The umbra is the darkest part of the shadow, where the light source is completely blocked by the occluding body. An observer in the umbra experiences a total eclipse. The penumbra is the region in which only a portion of the light source is obscured by the occluding body. We considered several issues, *i.e.*, the characteristic analysis of moving objects and shadows in different color spaces, shadow modeling and classification, a shadow elimination operator, and moving region detection.

Commonly used well-known color spaces include RGB, YIQ, YCrCb, XYZ, Luv, and HSV. Illumination invariants include hue (H), saturation (S), normalized *rgb*, c1 c2 c3, and l_1_ l_2_ l_3_ [15]. Our method for shadow removal is used the HSV model [16] and normalized the *rgb* to remove moving cast shadows. The first shadow mask (*FSD*) for each point (x, y) resulting from the object segmentation is defined as:

(1) $${\mathrm{FSD}}_{i}=\{\begin{array}{cc} 1 & \begin{array}{l} \mathrm{if}\;\;\alpha \leq \frac{I_{i}^{V}\;(x,\;y)}{B_{i}^{V}\;(x,\;y)}\;\leq \beta \;\;\wedge \;\;\left|I_{i}^{S}\;(x,\;y)-B_{i}^{S}\;(x,\;y)\right|\;\;\leq T_{S}\\ \wedge \;\left|I_{i}^{H}\;(x,\;y)-B_{i}^{H}\;(x,\;y)\right|\;\;\leq T_{H},\;\;\alpha \in \;[0,1],\;\beta \in \;[0,1] \end{array}\\ 0 & \mathrm{otherwise} \end{array}$$

where *I* and *B* represent the current image and a reference image as the background, respectively. *T_S_* and *T_H_* are the thresholds for limiting shadows. The threshold values used are empirically determined constants.

The two values r and g in the normalized rgb invariant color features are obtained as follows:

(2) $$r=\frac{R}{R+G+B}\;\;\;;\;\;g=\frac{G}{R+G+B}$$

where *R*, *G* and *B* are the intensity levels of red, green, and blue in the RGB color space, respectively. A small chromaticity change leads to a small change of *r*, *g*, or both of them because these values are independent. A pixel in the current frame is classified as shadow or shaded background if the pixel has a similar chromaticity but a lower luminance with respect to the corresponding the pixel in the reference image that is used as the background. To more accurately detect an object, the algorithm is utilized a second shadow mask (*SSD*). The *SSD* is conducted as follows:

(3) $${\mathrm{SSD}}_{i}=\{\begin{array}{cc} 1 & \begin{array}{l} \mathrm{if}\;\;\left(I_{i}^{R}\;(x,\;y)<\;B_{i}^{R}\;(x,\;y)\right)\;\wedge \;\left(I_{i}^{G}\;(x,\;y)<\;B_{i}^{G}\;(x,\;y)\right)\\ \wedge \;\left|I_{i}^{r}\;(x,\;y)-B_{i}^{r}\;(x,\;y)\right|-\left|I_{i}^{g}\;(x,\;y)-B_{i}^{g}\;(x,\;y)\right|\leq T_{n} \end{array}\\ 0 & \mathrm{otherwise} \end{array}$$

where *SSD* is set to 1 if the current pixel *i* is classified as shadow, and 0 otherwise. The proposed shadow removal method proceeds as follows. Let the current frame be *I_i_*

**Step 1**: Convert RGB model to HSV model and compute normalized r and normalized g from the RGB space.
**Step 2:** Verify if *I_i_*’s chromaticity is similar to the chromaticity of the background at the corresponding position in (1). If *FSD* is 1, then go to step 3, else the pixel *I_i_* belongs to the moving object of interest as the foreground.
**Step 3:** Verify if *I_i_*’s normalized r and g is in the range of shadows in (3). If yes, go to step 4, else *I_i_* belongs to a moving object as the foreground.
**Step 4:** *i* = *i* + 1. Repeat steps 1, 2, and 3 until all pixels in the extracted moving region are classified as shadow or shaded background. The algorithm records the trajectory and history information for each object.

**Figure 1.**
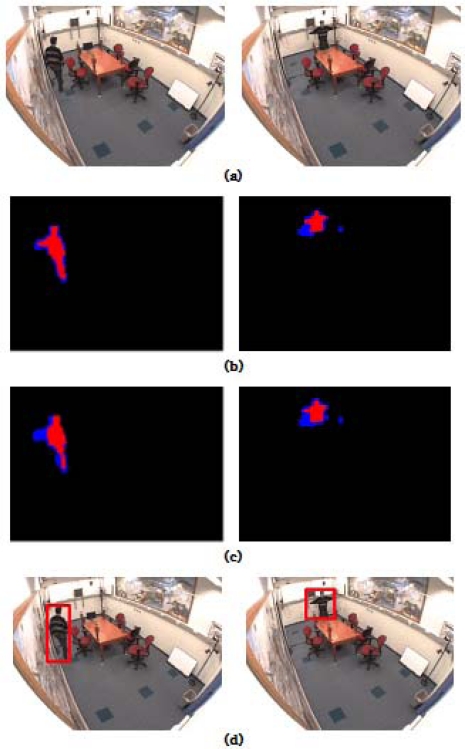
Segmentation results for image sequence “intelligent room”: (**a**) Original images; (**b**) Segmentation results with *FSD* using our shadow removal algorithm; (**c**) Segmentation results with *FSD* and *SSD* using our shadow removal algorithm; (**d**) Results of object detection.

To validate overall system performance, the proposed object detection with shadow removal is applied to a ground-truth dataset. The segmentation results for the image sequence “intelligent room” are shown in Figure 1. Figure 1(a) shows the original images captured from the “intelligent room” sequence that are used to evaluate the performance of our proposed shadow removal detection algorithm. Figure 1(b) shows the segmentation results with *FSD* for our shadow removal algorithm, while Figure 1(c) shows the segmentation results with a combination of *FSD* and *SSD* in order to remove moving shadow.

In Figure 1(b,c), the blue color represents shadows or noises derived from morphological operations or camera jitter. The red color indicates the detected moving object. The results for the combination of *FSD* and *SDD* shown in Figure 1(c) are more accurate than those in Figure 1(b). Figure 1(d) shows the results after detecting the object.

### 2.2. Moving Object Tracking

Moving object tracking is a significant problem in computer vision. There has been considerable recent interest in the analysis of videos containing humans. Human motion analysis is essential for a wide variety of applications, such as activity recognition, human-machine interaction, security surveillance, model-based compression, choreography, athletic performance analysis, and content-based retrieval.

We used an α–β–γ filter [17] to track a moving object of interest. The α–β–γ filter was particularly useful for tracking an accelerating target without steady-state error. When a new object appeared or each split object reappeared, the position of each moving object was predicted using the α–β–γ filter. The α–β–γ filter predicted the position of the bounding box for the object and matched it with the closest target foreground. The proposed system records about feature information of a target object of interest during tracking the subject. The important features include the object ID, MBR, position, centroid, color, binary map, and 3-D depth image. This information is also used to determine whether an abnormal event has occurred or not.

### 2.3. Abnormal Event Detection

Several researchers have recently developed vision sensor-based systems for the detection of fall events. However, they provide no solutions for detecting a variety of unexpected falls, such as backward falls, forward falls, falls to the right, and falls to the left. Most previous methods using 2D features information are not sufficient for distinguishing falls from normal daily activities, such as sitting down rapidly. Therefore, visual sensor based abnormal event detection methods have many limitations in fall detection. To overcome these limitations, a more reliable and efficient vision sensor-based abnormal event detection based on human shape features analysis and 3-D depth information is presented in this paper.

If a fall occurs, the algorithm can differentiate normal daily activity from this abnormal activity. To discriminate between abnormal and normal daily activity, the algorithm exploits three features, including the ratio of the bounding box, normalized 2-D velocity variations from the centroids, and 3-D depth information. The 2-D vertical velocity is represented from the motion of the centroid of the subject’s silhouettes. The 3-D depth values of the depth image are obtained from the low-cost camera in real time.

To identify and detect an abnormal event, we consider the use of two detectors. The first detector is an unexpected fall event detector *UFED*^1^, as shown in Equation (4). If the ratio change of the minimum bounding rectangle (MBR) enclosing a moving object of interest is greater than a threshold value, then the system determines the incidence of a fall event. The MBR ratio is higher with a fall compared with other normal activities. Therefore, the system readily detects fall incidents. If *UFED*^1^ cannot exactly detect fall events, such as the forward fall or the backward fall, then we use a second detector *UFED*^2^ shown in Equation (5). Compared with falling to the side, forward falls and backward falls are difficult to detect because a single camera is limited by its FOV angle and 2D image. To overcome the problems, we utilized the *UFED*^2^ detector that considers the MBR ratio, the value of velocity, and the depth value obtained from a depth image:

(4) $${\mathrm{UFED}}^{1}\;\{\begin{array}{cc} \mathrm{Fall} & \mathrm{MBR Ratio}\;\geq {\mathrm{Th}}_{1}\\ \mathrm{goto}\;{\mathrm{UFED}}^{2} & \mathrm{otherwise} \end{array}$$

(5) $${\mathrm{UFED}}^{2}\{\begin{array}{cc} \mathrm{Fall} & \begin{array}{l} {\mathrm{Th}}_{2}\leq \;\mathrm{MBR Ratio}<{\mathrm{Th}}_{1}\\ \mathrm{AND}\;\left(\left|\mathrm{Velocity}\right|\geq {\mathrm{Th}}_{\mathrm{velocity}}\right))\\ \mathrm{AND}\left|\mathrm{Variation of}\;3D\;\mathrm{depth value}\right|\geq {\mathrm{Th}}_{\mathrm{depth}} \end{array}\\ 0 & \mathrm{otherwise} \end{array}$$

## 3. Experimental Results

The system has been implemented and tested on an Intel Dual2core 2.2 GHz PC with 2.0 Gbyte of main memory, which operated in Microsoft Windows XP. The test video sequences, including color and depth, are captured in AVI format using a stationary camera at a frame rate of 30 frames per second and with a pixel resolution of 640 × 480.

The proposed algorithm is resized images to reduce time consuming. We applied our algorithm to a database containing different human activities. These activities included walking, standing, crouching down, standing up, falling forward, falling backward, falling to the right, and falling to the left. A total of 175 video activity sequences were captured in indoor environments using a Kinect sensor connected to a laptop computer. The Kinect consists mainly of a horizontal sensor bar which is composed of a RGB camera, depth sensor and a microphone.

**Figure 2.**
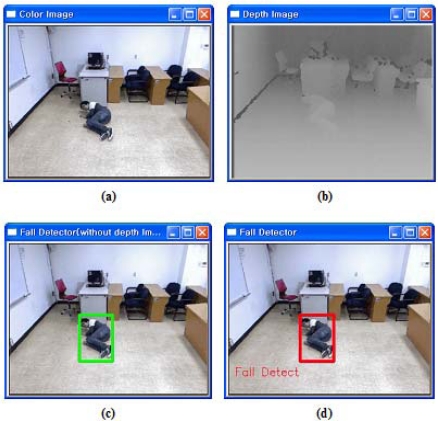
Results of abnormal event detection: (**a**) Original image; (**b**) Depth image corresponding to (a); (**c**) Result with *UFED*^1^ (not detected); (**d**) Result with a combination of *UFED*^1^ and *UFED*^2^ (detected).

The results of backward fall detection abnormal activities are shown in Figure 2. As shown in Figure 2(c), *UFED*^1^ could not detect the backward fall event. Figure 2(d) shows the successful detection of a fall event. The detected and tracked subjects are marked by a rectangle on the output frame. When a fall occurs, the system is displayed “Fall Detect” on the screen.

**Figure 3.**
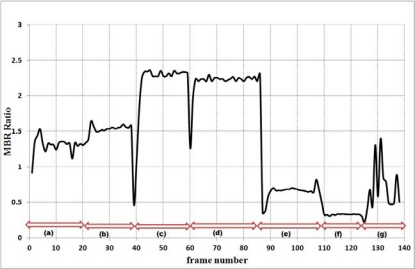
Variations in the MBR ratio: (**a**) Backward fall; (**b**) Forward fall; (**c**) Falling to the left; (**d**) Falling to the right; (**e**) Crouching down and standing up; (**f**) Standing; (**g**) Walking.

**Figure 4.**
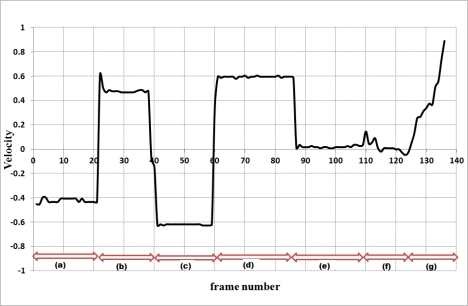
Variations of 2-D vertical velocity during different human activities: (**a**) Backward fall; (**b**) Forward fall; (**c**) Falling to the left; (**d**) Falling to the right; (**e**) Crouching down and standing up; (**f**) Standing; (**g**) Walking.

Results of our proposed abnormal event detection algorithm are depicted in Figures 3 and 4. The MBR ratios during the falls are obviously higher than the MBR ratios of normal daily activities as shown in Figure 3. If the first detector could not detect the fall, the second detector considers the velocity values and normalized depth value of abnormal activities.

**Figure 5.**
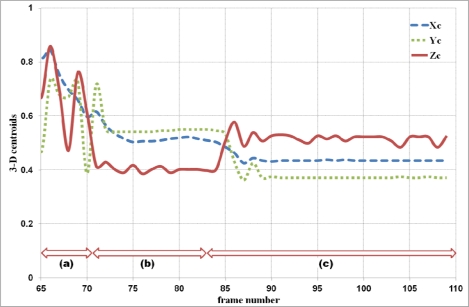
Variations of 3-D centroids for the example shown in Figure 2 during different human activities: (**a**) Walking; (**b**) Standing; (**c**) Backward fall.

When the ratios of a backward fall are below 1.5 for the example shown in Figure 2, the first detector UFED^1^ cannot detect the backward fall. Thus, the second detector UFED^2^ in our proposed algorithm can allow detecting the backward fall. Even though the MBR ratios for this example are lower than 1.5, the values of velocity for abnormal activities are greater than 0.3 or less than −0.3 during a certain time interval in Figure 4. We also considered 3-D depth values captured by a depth camera. The depth value can be decreased or increased during forward falls or backward falls. The normalized depth value (Z_c_) is increasing during the backward fall. After the fall is happened, the values are continuously over 0.5 in a certain time interval. Figure 5 illustrates variations of 3-D centroids of test image sequence in Figure 2. Centroid (X_c_, Y_c_, Z_c_) is the center of normalized mass coordinates of the person’s silhouette.

The normalized depth value is increasing when backward fall is happened. Thus, we used this feature information to differentiate abnormal and normal daily activity in our system to detect more accurate falls. The experimental results of our abnormal event detection algorithm with test videos in an indoor environment are shown in Table 1. The performance of the proposed system is shown in Table 2.

We measured sensitivity and specificity to evaluate system performance. Two main criteria are widely used in fall detection algorithms. A useful system has a high true positive proportion when it has a low false-positive proportion. This system provides high sensitivity and high specificity. Sensitivity is defined as the capacity of the system for correctly identifying true abnormal events, while specificity is defined as the capacity of the system for not generating false positives.

These values were calculated and expressed as follows: sensitivity was equal to the number of true positives/(number of true positives + number of false negatives) × 100; specificity was equal to the number of true negatives/(number of false positives + number of true negatives) × 100. True positives were the number of falls correctly detected by the system. False negatives were the number of undetected and misclassified falls. True negatives were the number of normal daily activities correctly detected by the algorithm, which were not true falls. False positives were the number of events falsely detected as fall. The detection rate of the hybrid human fall detection scheme [15] is about 91%. This scheme is quite lower than our proposed method. Additionally, the proposed abnormal event detection algorithm is more efficient and reliable than other falling detection studies in Section 1. The experimental results showed that the success rate of detecting abnormal events was 97% with a false positive rate of 2%. Our system provides a sensitivity of 94% and a specificity of 98%. Our system can allow discriminating diverse fall activities such as forward falls, backward fall, and falling asides from normal activities.

## 4. Conclusions

The detection of abnormal events, such as falls, is an important issue for elderly people when maintaining the quality of life and the independence of aged residents in residential care facilities. The primary goal of our research is to design a novel abnormal event detection system that can discriminate between abnormal activities and normal daily activities based on shape features analysis and the 3-D trajectory. An efficient and accurate algorithm for moving object segmentation and shadow removal is developed to improve the accuracy of detecting and tracking objects. Shadow removal is a major problem in many vision-based automated applications. Moving cast shadows can be misclassified as object parts or moving objects during moving object detection and in tracking processes, because the cast shadow region is included in the moving object region after the segmentation process. We validated the overall system performance of the proposed object detection and shadow removal algorithm by applying it to ground-truth datasets. To discriminate between abnormal and normal activities, the algorithm exploits three features, including the ratio of the bounding box, normalized 2-D velocity variations from the centroids, and 3-D depth information. The proposed visual sensor-based abnormal event detection algorithm is evaluated the effectiveness using a variety of test video sequences containing normal activities and simulated fall activities. Experiment results show very promising results for the proposed method using the low-cost USB camera.

## Figures and Tables

**Table 1.** Experimental results with our test videos.

**Truth**	**Abnormal activity**	**Normal activity**
**System detection**		
**Abnormal activity**	True Positive: 50	False Positive: 2
**Normal activity**	False Negative: 3	True Negative: 120

**Table 2.** System performance summary.

**Measure**	**Rate(%)**
Sensitivity	94%
Specificity	98%
False positive rate	2%
Accuracy	97%
